# Morbid Anatomy of the Fallopian Tubes

**Published:** 1887-08

**Authors:** 


### THE MORBID ANATOMY OF THE FALLOPIAN TUBES.

An article descriptive of the morbid conditions in twenty-five cases of tubal disease operated upon by Prof. Martin is contributed to the current issue of Virchow's *Archiv* by Dr. E. G. Orthmann. It should be read with interest at this time, when so much is being said regarding the clinical aspect of the chronic inflammatory changes to which the uterine appendages are prone. Dr. Orthmann describes in detail the histological characters of his specimens, and in the first place draws attention to the normal structure of the fallopian tube. The wall of the tube is composed of three layers—the mucous membrane, which, covered with ciliated epithelium, is disposed in longitudinal folds, which towards the free extremity become branched and divided, ultimately forming the so-called fimbriæ; the muscular layer, greatly developed in the uterine segment of the tube, and diminishing in thickness towards the abdominal extremity; and the outermost connective-tissue layer. The morbid conditions described fall under five heads, and each case is related in full. Of *catarrhal salpingitis* there were nine cases, six being unilateral. The disease is not limited to the mucous membrane, nor does the ciliated epithelium at first suffer, the change commencing with thickening of the mucous membrane, and consequent irregular swelling of its folds. The swollen folds coming into contact, the cells are compressed, degenerate, and disappear. More or less cystic cavities form between the thickened folds of mucous membrane; and blood-vessels in the infiltrated submucosa may rupture, giving rise to hæmorrhage into the cavity of the diseased tube. Such hæmatosalpinx may result from a closure of the fimbriæ. The muscular and fibrous coats of the wall of the tube may also be thickened, often with atrophy of the muscular fibers themselves. In five cases the "ostium abdominale" was occluded, partly by hypertrophy of the mucous folds, partly by adhesive peritonitis. The following varieties of catarrhal salpingitis are enumerated, the term "catarrhal" being obviously used in a wide sense:—(1) Salpingitis cat. simplex or endosalpingitis; (2) S. diffusa or interstitialis, which comprises (*a*) S. hæmorrhagica and (*b*) S. follicularis. Of *purulent salpingitis* eight cases are described. In seven of these the most careful examination failed to detect any bacterial organisms—a negative result which suggests either that the organisms were either remarkably scanty (as usual in chronic suppuration), or else that they could not be manifested by the methods used; reference, however, is made to the discovery by other observers of tubercle bacilli and of gonococci in tubercular and gonorrhœal salpingitis, and Dr. Orthmann himself has quite recently recorded an instance of the last named variety.* In purulent salpingitis only traces of the ciliated epithelium remain where protected by the folds which are now converted into wide septa between cavities filled with pus; whilst the mucous membrane is more or less replaced by the granulation cells of the inflammatory process. In places the muscular coat may be laid bare by the destructive process. This coat may also be thickened by infiltration of round cells, and the vessels will be increased. In six cases the ostium abdominale was occluded, and of the others one had a fistulous communication with an ovarian abscess. There were two cases of *hæmatosalpinx*, in one of which there was atresia of the ostium abdominale, the mucous folds were stretched, and the walls somewhat atrophied. In the other case the tube was occluded by a kink. Four cases of *hydrosalpinx* are described, three of which were unilateral. In all the supeficial epithelium was not much altered; in one case the mucous folds were hypertrophied, but in the rest they were dwindled, and the muscular coat was also atrophied. Lastly, there were two cases of *pyosalpinx*, a condition generally dependent upon atresia of the tube. The walls were thickened and their vascular supply increased. No microbes could be found in the purulent contents. Dr. Orthmann also gives a summary of these cases, from which we gather that in the twenty-five operations there were three deaths—one from collapse and two from peritonitis. Of these cases twenty-one were simple salpingotomies (two deaths). In two instances the patient underwent a second laparotomy. In one a right hydrosalpinx was removed seven months after the extirpation of the left appendages; in the other, a large hæmatoma of the right ovary had been removed thirteen years before. The majority of the patients had presented no disturbance of menstruation; in five cases it had been of late somewhat profuse; in five there was severe dysmenorrhœa, and in three menstruation was irregular. The chief and universal symptom was pain in the abdomen and groin, whilst in ten cases there occurred hæmorrhage, and in seven marked uterine discharge. As to the etiology of the cases, ten were ascribed to gonorrhœa, these including six purulent cases, two catarrhal, and the two cases of hydrosalpinx. Four were postpuerperal, but in the remainder no definite cause could be assigned. Two cases were associated with cancer of the uterus, one with myoma, and one with an ovarian cyst. The ovary was found diseased in all but two of the cases. In eight there was chronic ovaritis, two being further complicated with hæmatoma of the ovary and one with hydrops folliculorum. In four other cases there was ovarian hæmatoma, and in three others hydrops folliculorum, without other lesions. There were five cases of ovarian abscess, which in two cases communicated with the tube by small openings, and in one the tube and ovary were conjoined in a common abscess sac. One case of catarrhal salpingitis was associated with an ovarian cyst of the size of the fist. Atrophy of the ovary occurred three times, and parovarian cysts in four cases; in three cases there was hæmatoma between the ligaments, and in one an abscess. We have called attention to this contribution because it seems to us that the pathology of these conditions has yet to be worked out, whilst the material is rapidly accumulating for a thorough study of the subject.

*Vide Berl. Klin. Woch., No. 14, 1887.

